# Current Progress of Severe Fever with Thrombocytopenia Syndrome Virus (SFTSV) Vaccine Development

**DOI:** 10.3390/v16010128

**Published:** 2024-01-16

**Authors:** Dokyun Kim, Chih-Jen Lai, Inho Cha, Jae U. Jung

**Affiliations:** 1 Cancer Biology Department, Infection Biology Program, Lerner Research Institute, Cleveland Clinic, Cleveland, OH 44195, USA; kimd9@ccf.org (D.K.); laic3@ccf.org (C.-J.L.); chai@ccf.org (I.C.); 2 Global Center for Pathogen and Human Health Research, Lerner Research Institute, Cleveland Clinic, Cleveland, OH 44106, USA

**Keywords:** SFTSV, vaccine, emerging virus, tick-borne disease

## Abstract

SFTSV is an emerging tick-borne virus causing hemorrhagic fever with a case fatality rate (CFR) that can reach up to 27%. With endemic infection in East Asia and the recent spread of the vector tick to more than 20 states in the United States, the SFTSV outbreak is a globally growing public health concern. However, there is currently no targeted antiviral therapy or licensed vaccine against SFTSV. Considering the age-dependent SFTS pathogenesis and disease outcome, a sophisticated vaccine development approach is required to safeguard the elderly population from lethal SFTSV infection. Given the recent emergence of SFTSV, the establishment of animal models to study immunogenicity and protection from SFTS symptoms has only occurred recently. The latest research efforts have applied diverse vaccine development approaches—including live-attenuated vaccine, DNA vaccine, whole inactivated virus vaccine, viral vector vaccine, protein subunit vaccine, and mRNA vaccine—in the quest to develop a safe and effective vaccine against SFTSV. This review aims to outline the current progress in SFTSV vaccine development and suggest future directions to enhance the safety and efficacy of these vaccines, ensuring their suitability for clinical application.

## 1. Introduction

Severe fever with thrombocytopenia syndrome virus (SFTSV) is an emerging tick-borne virus leading to a viral hemorrhagic fever with a case fatality rate that can reach up to 27% [1]. Although the virus was renamed from *Huaiyangshan banyangvirus* to *Dabie bandavirus* and then *Bandavirus dabieense* by the International Committee on Taxonomy of Virus (ICTV) recently [2], we refer to it as SFTSV since it has been more widely described as such in the scientific community. The virus was originally identified in China in 2009 [3] and is now endemic to Japan, South Korea, and China with annual outbreaks [4]. Recent studies have also reported SFTSV infection in Vietnam, Thailand, and Taiwan, suggesting a potential expansion of endemic SFTSV infection areas [5,6]. Patients undergo an incubation period of 7 to 14 days after the initial infection. In the first stage of SFTS—the fever stage—the patients suffer from acute high fever, fatigue, viremia, thrombocytopenia, leukopenia, and lymphadenopathy. The second stage—multiorgan failure stage—is characterized by symptoms of elevated activated partial thromboplastin clotting time (aPTT), aspartate transaminase (AST), alanine transaminase (ALT), lactate dehydrogenase (LDH), exacerbated thrombocytopenia and leukopenia, neurologic symptoms, and disseminated intravascular coagulation (DIC) [7,8,9,10,11,12]. The SFTS prognosis, whether it leads to convalescent or a fatal infection, has been shown to be associated with the severity of cytokine storm characterized by the increase of IL-6 and IL-10 levels and the decrease of the transforming growth factor-β (TGF-β) level, as well as the alterations of B cell subpopulations attributed to B cell class switching [13,14,15,16,17]. Most importantly, fatally infected patients fail to mount an IgM and IgG response against SFTSV due to impaired humoral immunity [18,19,20,21].

Age has been identified as a crucial determinant for influencing the outcome of SFTSV infection, as the majority of morbidities and mortalities are focused on age groups of 50 or above and case fatality rate increases with age [1,22]. Considering suboptimal vaccine efficacy from immunosenescence, developing an effective vaccine to protect the older population from SFTSV infection and subsequent pathogenesis requires a vaccination approach with a high safety profile and immunogenicity [23,24].

With a lack of specific antiviral treatment with consensus on its efficacy, current therapeutic options to treat SFTS heavily rely on supportive care. Ribavirin (brand name Moderiba) has shown mixed reports in its efficacy, and the drug was mainly effective against patients in the earlier stage of infection with viral loads less than 1 × 10^6^ viral copies/mL [25,26,27,28]. Favipiravir (T-705, brand name Avigan) has shown broad antiviral efficacy against RNA viruses, such as influenza virus [29,30], Ebola virus (EBOV) [31], rabies virus [32], West Nile virus [33], and bunyaviruses [34,35,36], with some success in reducing fatality in a non-randomized uncontrolled trial. However, its therapeutic potential was also limited to younger patients who were treated at earlier stages of SFTS with low viremia, and the drug required a longer treatment period [26,34,35,36]. Considering the fatality rate is already significantly low in the younger population infected with SFTSV, its therapeutic efficacy in treating fatal SFTSV infection remains controversial. Therefore, the current treatment protocol places an emphasis on therapeutic plasma exchange, one of the most effective protocols to this date, to reduce viral load and inhibit the cytokine storm that leads to multiorgan failure [37,38,39].

Although there are accounts of nosocomial human-to-human transmission from infected individuals to healthcare providers and sporadic animal-to-human transmission [7,40,41], the primary route of infection is through a tick bite by an Asian longhorned tick (*Haemaphysalis longicornis*) [42,43,44]. The Asian longhorned tick functions as a vector and reservoir of SFTSV, maintaining the virus in the tick population via transovarial and transstadial transmission [45]. SFTSV has been identified in both the midgut and salivary glands of the tick, leading to transmission to human or vertebrate animals upon blood feeding [45,46]. However, the duration and severity of SFTSV viremia in vertebrate animals other than humans are limited despite the high seroprevalence of SFTSV in field studies of wild animals, suggesting exposure to the virus [47,48,49]. The recent spread of SFTSV to Vietnam, Thailand, and Taiwan is also attributable to the spread of the tick, as shown in the distribution of the Asian longhorned tick matching the East Asian–Australian flying routes of migratory birds [50].

Due to climate change, there has been an expansion of the potential habitat of ticks to wider geographical locations, such as North America, Australia, and New Zealand, posing a risk of introducing the exotic tick species that may carry SFTSV [44,51,52,53,54]. Recent findings of the tick in over 20 states in the United States correspond with the growing concern regarding SFTSV as an imminent public health issue [55,56]. Its parthenogenesis of asexually laying approximately 2000 eggs maturing in 2 weeks is expected to accelerate the dissemination of the tick further [42,57]. Correspondingly, SFTSV has recently been designated as a priority pathogen by the World Health Organization (WHO) [58] and the United States National Institute of Allergy and Infectious Diseases (NIAID) [59]. With the lack of licensed vaccines or targeted therapies against SFTSV currently, there is a growing risk of the SFTSV outbreak beyond East Asia and the increasing necessity of research interest to develop safe and effective vaccines against SFTSV.

This review primarily focuses on describing the biology and immunogens of SFTSV to develop effective vaccines. Additionally, this review aims to illustrate animal models replicating human SFTSV infection and pathogenesis to study the protective immunity of the vaccine candidates. Finally, this review provides a detailed analysis of the current SFTSV vaccines developed, assessing their strengths and weaknesses concerning potential human clinical application.

## 2. SFTSV Biology

SFTSV is classified within the *Bandavirus* genus of the family *Phenuiviridae* within the order *Bunyavirales* [2]. The virus carries three single-stranded RNA segments: two of which are negative-sense, and one is ambisense [3]. Each of the three segments of the viral genome, L, M, and S, encodes L protein, glycoproteins Gn and Gc, and nucleoprotein (N) and nonstructural protein (NSs), respectively [3]. L protein comprises cap-binding domain, endonuclease domain, and RNA-dependent RNA polymerase (RdRp) enzymatic domain for host mRNA 5′ cap-snatching, as well as viral transcription and genomic replication during the viral lifecycle [60]. Glycoproteins are translated as a single precursor, then cleaved to Gn and Gc by host proteases to form a virion approximately 100 nm in diameter [61,62,63,64]. N forms hexamers and interacts with viral RNA (vRNA) to make viral ribonucleoprotein (RNP) to protect the vRNA from degradation by filling the internal virion space [63,64,65]. NSs is a major pathogenic protein that sequesters the host protein complex of tumor progression locus 2 (TPL2)/A20-binding inhibitor of NF-κB activation 2 (ABIN2)/p105 into an inclusion body to induce IL-10 secretion and subsequent anti-inflammatory signaling [66,67].

Structural glycoproteins forming viral envelopes and carrying neutralization epitopes have been considered promising vaccine antigens, as demonstrated in SARS-CoV-2 [68,69,70,71], influenza virus [71], and EBOV [72]. A recent cryo-electron microscopy (cryo-EM) has elucidated the structural importance of Gn and Gc glycoproteins in forming the viral lipid bilayer envelope. Gn forms a heterodimer with pre-fusion Gc, and then 12 heterodimers form three different hexon and penton formations. The hexons and pentons ultimately assemble into an icosahedral virion shell comprising 720 Gn-pre-fusion Gc heterodimers [63,64]. During the viral infection, Gn is presumed to attach to the putative host cell receptor C-C motif chemokine receptor 2 (CCR2) to mediate viral entry [73]. Followed by receptor-mediated endocytosis of the virion particle, Gc mediates membrane fusion between the host cell membrane and the viral envelope to release vRNA into the host cell cytoplasm [61,62,63,64]. In line with the role of glycoproteins in the viral life cycle, recent studies of SFTSV-neutralizing antibodies from convalescent human sera have reported the Gn head region as the neutralization epitope [74,75,76]. Most successful vaccine development approaches described here also encompass glycoproteins to support their immunogenic potential further.

Besides the rational selection of viral proteins, the SFTSV diversity across the six genotypes (A–F) must also be considered to develop an effective vaccine that provides as much broad protection as possible [1,50,77,78,79,80]. Different geographic locations have significant variations of case fatality rates, such as 5.3 to 16.2% in China, 23% in South Korea, and 27% in Japan, attributed to different SFTSV genotypes dominating the countries [1]. Furthermore, SFTSV readily undergoes reassortments due to the segmented viral genome [1,81,82]. Much of the literature has reported reassortants rising in various geographic locations: nine or more in South Korea and seven or more in China [1,4,78,79,83]. The lack of proofreading activity of the SFTSV RdRp protein also suggests a potential increased risk of variants, complicating successful vaccine development [84]. However, variations of the M segment, which encodes the Gn and Gc glycoproteins, across the genotypes have been limited to less than 10% of the viral nucleotide and 6% of the amino acid sequence, which suggests potential cross-genotype protection of SFTSV vaccines developed as of today [85]. Considering these, an ideal SFTSV vaccine should prevent initial infection and SFTS pathogenesis, with broad protection at a low cost for accessibility.

## 3. Animal Models to Study Efficacy of SFTSV Vaccines

Although widely used for in vivo studies, the application of wild-type (WT) mouse models is limited in SFTSV vaccine development. WT BALB/c, CD-1, and C57BL/6 mouse models demonstrate a brief viremia in the spleen and mild thrombocytopenia and leukopenia after SFTSV infection. However, the virus is rapidly cleared due to the failure of viral replication in WT mice. Furthermore, the WT mouse models fail to replicate fatal SFTSV infection accompanying fever, severe body weight loss, and multiorgan failure [86,87]. Currently, available animal models developing SFTSV infection and SFTS symptoms are confined to immunosuppressed animals and aged ferrets [87].

### 3.1. Newborn Mouse

In a previous study, newborn (one-to-three days old) WT C57BL/6 mice were infected with different titers of SFTSV, ranging from 6 × 10^5^ to 6 × 10^8^ SFTSV titers [88]. Animals infected with 6 × 10^5^ copies demonstrated 58% fatality by 17 days post-infection (dpi), while those infected with higher titers of 6 × 10^6^ or 6 × 10^8^ viral copies showed 100% fatality by 17 dpi. However, newborn mice have not developed a complete immune system and, thus, are limited in recapitulating human SFTSV infection and subsequent age-dependent pathogenesis. Most importantly, vaccination protocols often require multiple weeks for several doses and the activation of adaptive immunity, which limits the application of newborn mouse models for SFTSV vaccine development studies.

### 3.2. Type I interferon Receptor (IFNAR) KO Mouse

The IFNAR KO mouse model has been extensively employed to investigate the pathogenesis and vaccine-induced protective immunity against EBOV [89], Zika virus (ZIKV) [90], and Crimean-Congo hemorrhagic fever virus (CCHFV) [91], highlighting the immunological importance of type I interferon signaling pathway in antiviral defense. Previous studies have demonstrated clinical symptoms of IFNAR KO mice infected with various titers of SFTSV (between 10^2^ and 10^5^ TCID_50_) through intradermal, intraperitoneal, intramuscular, or subcutaneous injection [36,89,92]. IFNAR KO mice injected with 10^5^ TCID_50_ demonstrated significant body weight loss of up to 30%, serum viremia, thrombocytopenia, and 100% fatality by six dpi regardless of the routes of infection [87]. Infected mice also demonstrated the disruption of lymphoid follicles and sinuses in the spleen and lymph nodes, the depletion of erythroid precursor cells and necrosis in bone marrow, hepatocytic necrosis, and disrupted the liver structure to replicate the multiorgan failure of human SFTSV. Viral N antigen was detected from the spleen, lymph node, liver, and kidney to suggest systemic viremia in the infected IFNAR KO mice [87,92]. Although the SFTSV pathogenesis pattern, like that of human SFTSV infection, supports the efficacy of the IFNAR KO mouse model for SFTSV vaccine development study, the lack of IFNAR signaling pathway is a concern in replicating immune activation in healthy individuals immunized with SFTSV vaccine candidates [85,87,93].

### 3.3. WT Mouse Treated with IFNAR-Neutralizing Antibody (IFNAR-NAb)

To address the concern of immunosuppression from the lack of an IFNAR signaling pathway, a plethora of virus research replaced the IFNAR KO mice by injecting WT mice with IFNAR-NAb for one or two days prior to infection. Common protocols inject IFNAR-NAb (clone MAR1-5A3) once with 1 μg at 24 h or twice with 0.5 μg at 48 and 24 h prior to SFTSV infection to suppress IFNAR signaling [93,94]. Clinical manifestations characterized as SFTSV viral load in the serum, spleen, lymph node, bone marrow, and kidney from IFNAR-NAb-treated mice have been reported to be similar to those of IFNAR KO mice. However, the lethal dose for WT mice treated with IFNAR-NAb has been reported to be higher than that of IFNAR KO mice, complicating the use of this model in SFTSV vaccine development. Furthermore, a potential batch effect of the IFNAR-NAb leading to insufficient or excessive IFNAR signaling neutralization is the most prominent obstacle in testing various combinations of antigens and their doses and adapting this animal model for SFTSV vaccine development [93,94].

### 3.4. Non-Human Primates (NHP)

Due to the resemblance of NHP’s immune system to the human immune system, NHP has been considered the ideal model to study the wide range of viral pathogenesis and vaccine development, as shown in Epstein-Barr virus (EBV) [95,96], human immunodeficiency virus-1 (HIV-1) [97], and hepatitis C virus [98]. In addition, NHP models, such as cynomolgus macaque and rhesus macaques, have been used to study bunyaviruses that cause viral hemorrhagic fever, including Crimean-Congo hemorrhagic fever virus, arenaviruses, and hantavirus [99,100,101,102,103,104]. However, cynomolgus macaques [87] and rhesus macaques [105] subcutaneously infected with SFTSV 10^6^ TCID_50_ showed no visible clinical signs or mild illness in some infected animals. The animals also did not induce viremia upon infection at 1, 3, 5, 8, 11, and 14 dpi, limiting the usage of NHP in SFTSV vaccine development [87]. 

### 3.5. Aged Ferret

The predominant factor influencing the disease outcome of SFTSV infection is age, as indicated by the majority of fatal cases focused on age groups of 50 or above; the elderly population also contributes to the high case fatality rate of up to 27% to support the trend [12,18,22]. Therefore, the SFTSV vaccine development approach requires an animal model recapitulating age-dependent pathogenesis and clinical outcome. Park et al. reported an age-dependent ferret (*Mustela putorius furo*) model that replicates the disease progression and symptoms of human SFTSV infection [106]. Aged ferrets (4 years or older, equivalent to 70 years old for a human) demonstrated severe fever, body weight loss, thrombocytopenia, leukopenia, and serum and organ viremia, and 93% fatality by six dpi upon infection with 10^7.6^ TCID_50_ SFTSV. By contrast, young adult ferrets (2 years or younger, equivalent to 30 years old human) did not demonstrate any symptoms. Further transcriptomic analysis of infected aged ferrets suggested well-known antiviral genes, such as C-X-C-motif chemokine 10 (CXCL10), 2′-5′-oligoadenylate synthetase-like (OASL), and IL-5 receptor-α, as the most differentially expressed genes (DEGs). Although the aged ferret model provides the best replication of immune activation upon vaccination and pathogenesis from SFTSV infection, the lack of resources to study ferret immunology, limited supply of aged ferrets, and dedicated housing facility of Animal Biosafety Level-3 (ABSL-3) for ferrets limit the usage of the aged ferret model to study SFTSV vaccine development [87].

## 4. Types of SFTSV Vaccines Developed to Date

### 4.1. Live-Attenuated Vaccine Platform

Live-attenuated vaccines can trigger highly potent immunogenicity. However, it has a drawback when used against the immunocompromised population: the live-attenuated virus could undergo mutation to become virulent or elicit excessive immunogenicity [107,108,109]. 

Viral NSs protein was previously reported as a major virulence factor of SFTSV by antagonizing the TPL2 signaling pathway and IFN-β secretion to evade induction of an antiviral state [66,67]. Two recombinant viruses carrying mutations in NSs have been shown to fail to antagonize the TPL2 and IFN-β pathways [66,110], suggesting their potential as live-attenuated vaccines (Table 1). Yu et al. generated rHB2912aaNSs deletion mutant—carrying only the first methionine residue and the last 11 amino acids of NSs—and rHB29NSsP_102_A point mutant—carrying a proline to alanine mutation—viruses via a reverse genetics system [111]. To test for potential vaccine candidates’ virulence, the greatest concern of the live-attenuated vaccine development approach, Yu et al. [111] immunized aged ferrets with 4 × 10^6^ plaque-forming units (PFU) of rHB2912aaNSs, 5 × 10^5^ PFU of rHB29NSsP_102_A, or 5 × 10^5^ PFU of WT pathogenic strain SFTSV (CB8/2016). Aged ferrets injected with the CB8/2016 strain showed severe body weight loss, fever, serum viremia, and 100% fatality by 12 dpi. However, animals immunized with the two live-attenuated vaccine candidates did not show the symptoms, indicating that the vaccine candidates do not induce pathogenesis in immunized animals.

Animals immunized with these two vaccine candidates induced potent humoral immunity upon immunization [111]. The titration of neutralizing antibodies from serum samples collected at 0, 8, 14, and 58 days after the single immunization demonstrated an increased neutralization titer quantified as a focus reduction neutralization test (FRNT_50_) until 14 days after the immunization. The titer remained high until 58 days after the immunization, and there was no significant difference in FRNT_50_ between rHB2912aaNSs and rHB29NSsP_102_A. Most significantly, the immunized animals were fully protected from body weight loss, fever, and fatality upon lethal viral infection with 10^7.6^ TCID_50_ of SFTSV at eight weeks after the immunization. Furthermore, aged ferrets previously immunized with the live-attenuated vaccines were fully protected from serum viremia. When performing quantitative real-time PCR (qRT-PCR) on the S segment of SFTSV RNAs to determine the viral RNA copy number, the viral RNA level was under the detection limit in the immunized group until 12 days after the challenge. Cross-genotype protection of the two vaccine candidates developed based on genotype D (HB29 strain) also demonstrated immune protection against genotype B (CB1/2014 strain).

To test for potential reversion mutation of the rHB2912aaNSs strain to a pathogenic strain, the spleens of ferrets infected with the vaccine candidate were harvested at four dpi to isolate virus in Vero E6 cells. Upon qRT-PCR of splenic tissues after six rounds of passaging in ferrets, the deletion of NSs was intact without unexpected nucleotide mutation in the S segment to support the safety of rHB2912aaNSs as a live-attenuated vaccine candidate against SFTSV.

### 4.2. DNA Vaccine Platform

DNA vaccines have their merits in triggering strong immune responses, manipulating viral antigens for production, and having a low cost of production. However, there are concerns about using DNA vaccines, as DNA may integrate into the host genome or require an electroporator for immunization [107,108].

#### 4.2.1. DNA Vaccine Encoding Gn/Gc

As suggested by neutralization epitopes discovered primarily from the viral glycoprotein Gn, Kwak et al. focused on the M segment encoding Gn and Gc as a vaccine candidate for DNA vaccine development [112]. The consensus sequence of RdRp, Gn, Gc, N, or NSs from clinical isolates acquired from China, Korea, and Japan was cloned into a pVax1 expression vector. A weight of 200 μg of DNA expression vectors in PBS were electroporated to aged ferrets. Vectors encoding Gn and Gc (Gn/Gc) elicited the most robust cellular immunity, characterized as spot-forming units (SFU) in IFN-γ ELISpot of ex vivo-stimulated PBMCs two weeks after the final immunization. Gn/Gc DNA expression vectors also efficiently induced humoral immunity, as shown in the neutralization titer quantified as FRNT_50_ reaching 1 × 10^4^.

Aged ferrets immunized with the Gn/Gc DNA vaccine or PBS control were infected with a lethal dose of SFTSV (10^7.6^ TCID_50_) at 2 or 4 weeks after the final immunization. While DNA vaccine-immunized ferrets were fully protected from serum viremia, fever, thrombocytopenia, leukopenia, body weight loss, and fatality until 14 dpi, PBS control-immunized groups demonstrated a significant number of the above symptoms and 100% fatality by nine dpi. Aged ferrets that received serum transfer from other aged ferrets immunized with DNA vaccines encoding N, NSs, RdRp, or a combination of N/NSs/RdRp showed 100% fatality by 11 dpi. On the other hand, aged ferrets receiving serum from those immunized with a DNA vaccine encoding Gn/Gc were fully protected from the symptoms. These data highlight the efficacy of a DNA vaccine encoding Gn and Gc in protecting aged ferrets against lethal SFTSV infection.

#### 4.2.2. DNA Vaccine Encoding Gn/Gc and Mouse IL-12 (mIL-12)

In a different study developing a DNA vaccine, Kang et al. designed vectors encoding Gn, Gc, N, and NSs, with or without mIL-12; the function of mIL-12 is to enhance cellular immunity [113]. IFNAR KO mice were immunized with 4μg of the DNA vaccine via electroporation at weeks 0, 2, and 4. The DNA vaccine without mIL-12 failed to induce SFTSV N-recognizing IgG, indicating that mIL-12 is important for eliciting efficient immune activation. Upon lethal infection with 1 × 10^5^ focus-forming units of SFTSV, mice immunized with the Gn, Gc, N, and NSs DNA vaccine with mIL-12 showed full protection against fatality and mild protection against body weight loss, thrombocytopenia, and serum viremia. However, mice immunized with the Gn, Gc, N, and NSs DNA vaccine without mIL-12 demonstrated only 40% survival and pronounced body weight loss, thrombocytopenia, and serum viremia, characterizing insufficient immunogenicity to provide protective immunity against lethal infection. The different results in clinical outcomes from lethal SFTSV infection from Kwak et al. [112]—although both DNA vaccines include Gn and Gc—are attributable to the different doses and animal models used. The data indicate that DNA vaccine candidates require optimization to provide sufficient immune protection in immunized animals. However, including mIL-12 in the vaccine may potentially cause side effects in immunized animals and poses a concern in translation to humans.

### 4.3. Whole Inactivated Viral Vaccine Platform

Whole inactivated viral vaccines have their advantages in that the virus cannot undergo mutations to become virulent, which is safer to use in immunocompromised populations compared to live-attenuated vaccines, and they mimic natural infection, which can elicit balanced B and T cell activation [107,108]. However, the limitation is that this vaccine type is less immunogenic compared to live-attenuated vaccines, which may require several immunizations to elicit potent immunogenicity. In addition, it may be logistically difficult to perform a large culture of BSL-3 agents, such as SFTSV. 

Li et al. developed a whole β-propiolactone (BPL)-inactivated viral vaccine [114]. Supernatant from Vero cells infected with SFTSV were inactivated by BPL for 24 h and purified for virion particles via sucrose gradient. BALB/c and C57BL/6 mice were intramuscularly immunized with doses (0.25, 1, or 4 μg) of inactivated SFTSV based on Gn/Gc glycoprotein amounts with or without aluminum hydroxide Al(OH)_3_ adjuvant at weeks 0, 2, and 4. Mice immunized with the highest dose of 4 μg of inactivated SFTSV with Al(OH)_3_ induced the most robust IgG response against the SFTSV N and serum neutralization titer against the HB29 strain of SFTSV. On the other hand, anti-N IgG responses and serum neutralization titers were comparable across the rest of the doses with or without the adjuvant.

Immunized C57BL/6 mice were infected with 10^5^ TCID_50_ of SFTSV two weeks after the final immunization. The serum, liver, spleen, and kidney were collected at three and seven dpi to characterize serum and organ viremia. Although mice immunized with the highest dose of inactivated SFTSV with adjuvant showed the most significant acceleration in viral clearance, there was no statistical significance across different doses and adjuvant groups. However, since mouse models are limited to WT BALB/c and C57BL/6 that do not develop symptoms from SFTSV infection, the scope of the immunogenicity of the whole inactivated SFTSV vaccine is limited to the induction of humoral and cellular immunity. Further studies employing pathogenic animal models are needed to characterize the potential induction of protective immunity upon immunization with the whole inactivated SFTSV vaccine.

### 4.4. Viral Vector Vaccine Platform

Viral vector vaccines rely on harmless recombinant viral vectors, such as adenovirus or vesicular stomatitis virus (VSV), to deliver the target antigens for expression. As this vaccine platform utilizes the virus, it can mimic natural infection, trigger strong induction of the B and T cell response, and has advantages in mass production [108,109]. However, a potential drawback of viral vector vaccines is that the pre-existing immunity against the viral vectors can diminish the immunogenicity of the target antigens and poses a risk for side effects.

#### 4.4.1. Recombinant Vesicular Stomatitis Virus (rVSV)-Vectored Vaccine

rVSV has been widely used in vaccine development efforts against SARS-CoV-2 [115], CCHFV [116,117], and EBOV [72] due to VSV’s amenability by allowing an exchange of VSV glycoprotein (GP) with a heterogenous virus’ surface glycoproteins. Dong et al. developed rVSV expressing Gn/Gc from AH12 strain SFTSV (rVSV-SFTSV/AH12-GP) using the HEK293T cell line [118]. C57BL/6 and IFNAR KO mice were intraperitoneally immunized with a 2 × 10^4^ focus forming unit (FFU) of various rVSV-carrying glycoproteins from EBOV, Hantaan virus (HTNV), or SFTSV to test for the virulence of the vaccine candidates. The study showed that VSV or EBOV glycoprotein-carrying rVSV led to 100% fatality by three- and five-days post-immunization, respectively, and showed virulence in IFNAR KO mice. On the other hand, rVSV-SFTSV/AH12-GP, in which the incorporation of SFTSV/AH12-GP attenuated the growth of rVSV in culture, did not induce body weight loss or fatality in IFNAR KO mice.

Serum samples were collected from C57BL/6 mice 30 days post-immunization to quantify serum neutralization titer against SFTSV. Interestingly, mice immunized with rVSV-SFTSV/AH12-GP induced a neutralizing antibody response against AH12 and YG1 strains of SFTSV and Heartland virus (HRTV), a closely related Bandavirus [118,119]. Immunized IFNAR KO mice were challenged with 21 × 10^4^ FFU of SFTSV to characterize protective immunity against serum viremia, body weight loss, and fatality. Mice immunized with rVSV-SFTSV/AH12-GP or rVSV expressing HRTV Gn/Gc (rVSV-eGFP-HRTV-GP) were fully protected from symptoms when challenging with either SFTSV or HRTV, suggesting cross-protection. These findings suggest not only successful protection against lethal SFTSV infection in a single dose, but also the broad cross-protection of rVSV-SFTSV/AH12-GP and rVSV-eGFP-HRTV-GP vaccines against HRTV and SFTSV infection.

In a further study by Hu et al., two spontaneous mutations (M749T and C617R) were identified to improve Gc localization on the plasma membrane of the HEK293T cell during rVSV assembly and, ultimately, enhance the titer [120]. Immunogenicity characterized as serum neutralization titer (FRNT_50_) and protection against serum viremia, body weight loss, and survival was not altered upon the point mutations when the titer increased from 3 × 10^6^ to 7 × 10^7^ PFU/mL. This study is expected to enhance the logistics aspect of the rVSV-vectored SFTSV vaccine by reducing the cost of production.

#### 4.4.2. Recombinant Vaccinia Virus (rVACV)-Vectored Vaccine

Strains of Vaccinia virus (VAC) currently approved for vaccinating healthy individuals are attenuated modified vaccinia virus Ankara (MVA) and LC16m8 (m8) strains, which limit side effects while maintaining the immunogenicity as viral vectors [121] against EBOV [122] and Middle-East Respiratory Syndrome-Coronavirus (MERS-CoV) [123]. Yoshikawa et al. reported recombinant m8 vaccinia virus expressing SFTSV N (m8-N), glycoproteins without N (m8-GPC) and with N (m8-N+GPC) to form virus-like particles (VLP) [124]. IFNAR KO mice were subcutaneously immunized with 1 × 10^6^ PFU of m8-N, m8-GPC, or m8-N+GPC at weeks 0 and 2. All vaccine candidates effectively induced IgG recognizing SFTSV N protein or glycoproteins, but only m8-GPC and m8-N+GPC produced SFTSV-neutralizing titer without a significant difference. Upon lethal infection with 1 × 10^3^ or 1 × 10^5^ TCID_50_ of YG-1 strain SFTSV, the IFNAR KO mice were fully protected from body weight loss and fatality.

In a separate in vivo study, IFNAR KO mice were first immunized with Lister strain VACV, which is used to protect against smallpox virus, then immunized with the m8-N, m8-GPC, or m8-N+GPC to investigate the potential effect of pre-existing immunity against the viral vector vaccine. The results showed reduced protection to 30% survival (m8-N), 60% survival (m8-N+GPC), and 70% survival (m8-GPC) against the lethal SFTSV challenge of 1 × 10^5^ TCID_50_. These data suggest the rVACV-based vaccine provides protective immunity with a limitation in immunizing people born before the 1980s to have pre-existing immunity against VACV [124].

### 4.5. Protein Subunit Vaccine Platform

Protein subunit vaccines have been established for their high safety profile, providing immunity with little to no adverse effects upon immunization [107,108,125,126]. As SFTSV infection and prognoses mainly occur in elders who are immunocompromised, protein subunit vaccines provide merits for clinical application. However, these vaccine types also have the weaknesses of relatively weak immunogenicity attributed to inefficient antigen-presenting cells’ recognition and, subsequently, poor germinal center activation in lymph nodes [109,127]. To improve immunogenicity, adjuvants, such as MF59, have been included to provide robust immune responses [128,129]. Yet, a rational design of viral protein as a vaccine target and applications from biotechnology are still required to enhance immunogenicity to develop a safe and effective vaccine [130].

#### 4.5.1. Recombinant NSs Protein with Complete Freund’s Adjuvant (CFA)

NSs have already been reported as a major virulence factor of SFTSV. Liu et al. subcutaneously immunized WT C57BL/6 with 100 μg of SFTSV NSs purified from the bacterial expression system in combination with CFA at weeks 0 and 2 [131]. Immunized mice induced the antibody response against NSs, and ex vivo-stimulated splenocytes of immunized mice with the NSs peptides demonstrated a significant induction of cellular immunity characterized as IFN-γ. Immunized mice showed no symptoms upon challenge with 3 × 10^7^ PFU of SFTSV 12 days after the final immunization, but failed to accelerate viral clearance compared to the control group immunized with only adjuvant. Furthermore, CFA caused adverse effects by increasing platelet counts significantly and five-to-ten-fold in IL-2, -5, -6, IFN-γ, and TNF-α levels. This study clearly demonstrates a challenge in developing a safe and effective vaccine against SFTSV via a protein subunit vaccine approach.

#### 4.5.2. Gn and Gc-Fc

Previously described successful SFTSV vaccines encompass viral glycoproteins Gn and Gc. Kang et al. fused a human Fc fragment to SFTSV Gn (Gn-Fc) and Gc (Gc-Fc) for enhanced immunogenicity in IFNAR KO mice immunization [113]. IFNAR KO mice were immunized with 20 μg of purified Gn-Fc or Gc-Fc in combination with Al(OH)_3_ adjuvant at weeks 0, 2, and 4. Serum samples collected one week after the last immunization showed successful induction of anti-Gn/Gc antibody and serum neutralization titer against SFTSV infection. Although there was no difference in anti-Gn/Gc IgG titers, serum neutralization titer (FRNT_50_) was significantly higher (approximately 10-fold) with Gn-Fc immunization than Gc-Fc immunization. Surprisingly, both Gn-Fc and Gc-Fc vaccine candidates failed to provide sufficient protective immunity upon lethal SFTSV infection with 1 × 10^5^ FFU. All mice immunized with Gc-Fc succumbed to the fatal infection by eight dpi, and animals immunized with Gn-Fc showed 50% fatality by 10 dpi. Both groups demonstrated significant body weight loss of up to 20%. This study further shows the challenge of developing an effective protein subunit vaccine against SFTSV.

#### 4.5.3. Self-Assembling Gn Head-Ferritin Nanoparticle Vaccine

Although the protein subunit vaccine is considered the safest and most promising approach to develop a vaccine to protect the most susceptible population—elderly people—there is only one successful approach thus far. Kim et al. fused the head region of Gn (GnH) to a ferritin nanoparticle to develop a self-assembling GnH-ferritin nanoparticle (GnH-FT) [132]. The hybridization of bullfrog (*Rana catesbeiana*) and *Helicobacter pylori* ferritin to increase immunogenicity in the protein subunit vaccine has shown its potential against SARS-CoV-2 [133], EBV [96], and influenza virus [134,135]. The nanoparticles were expressed by transfecting HEK293T cells with plasmids encoding GnH-FT and purified by ion exchange chromatography and gel filtration. Purified GnH-FT nanoparticles were visualized by negatively stained transmission electron microscopy (TEM) and cryo-EM to observe the presence of GnH on the surface of the ferritin nanoparticle core.

BALB/c mice were intramuscularly immunized with 1, 5, or 10 μg of GnH-FT with AddaVax adjuvant, a veterinary equivalent of MF59 adjuvant, three times at weeks 0, 3, and 6. A weight of 3.3 μg of naked ferritin nanoparticle (FT) was included as a control antigen [132]. MF59 adjuvant is globally used in adjuvanted seasonal flu vaccines to immunize the elderly population due to its high safety profile and efficacy, which is an optimal adjuvant for a safe SFTSV vaccine for the elderly population. All mice immunized with GnH-FT induced IgG recognizing purified SFTSV GnH after the first and second immunization. The maximal anti-GnH IgG titers were achieved after the second immunization without future increase after the third immunization. However, serum neutralization titer continued to increase even after the third immunization. Ex vivo-stimulated splenocytes showed strong IFN-γ ELISpot and TNF-α and IL-2 intracellular cytokine production regardless of GnH-FT immunization dose. Interestingly, both humoral and cellular immunity were most potently induced from animals immunized with 1 μg, followed by 5 μg and 10 μg of GnH-FT nanoparticle.

The induction of GnH-recognizing and SFTSV-neutralizing antibodies was reproduced in the immunization of aged ferrets. Aged ferrets were immunized with 15 μg of FT or GnH-FT at weeks 0, 2, and 4, then challenged with a lethal titer of SFTSV (10^7.6^ TCID_50_) to characterize protective immunity and vaccine efficacy. Aged ferrets immunized with the control FT exhibited substantial body weight reduction of up to 20%, 100% fatality, and significant thrombocytopenia and leukopenia, reaching the lower limit of detection by eight dpi. By contrast, aged ferrets immunized with GnH-FT showed full protection from the symptoms with only mild fever at four dpi. Furthermore, ferrets immunized with FT showed significant serum viremia and constant increased viral loads in various organs (spleen, liver, and kidney), while those immunized with GnH-FT readily cleared the virus in serum and organs. These findings suggest that GnH-FT is a promising vaccine candidate against SFTSV, as the protein subunit vaccine is the safe vaccine approach. Nevertheless, additional investigation is necessary to clarify the mechanism of how the lowest 1 μg dose provides the most robust humoral and cellular immunity to establish a vaccination protocol that uses minimal effective doses to reduce adverse effects and production costs.

### 4.6. Heterologous Vaccination

Heterologous vaccination of immunizing more than one form of vaccine approach has shown its efficacy in concurrent activation of cellular and humoral immunity against HIV-1 [136], *Mycobacterium tuberculosis* [137], and *Plasmodium vivax* [138]. However, immunizing more than two vaccine types can complicate the immunization protocol. Kim et al. developed and tested the immunogenicity of two heterologous and two homologous vaccines by employing recombinant Adenovirus type 5 (rAd5) carrying Gn (rAd5-Gn) and purified Gn protein [139]. The Gn protein vaccine was adjuvanted with Al(OH)_3_ and CrPV mRNA adjuvants. Six-month-old WT C57BL/6 mice and eighteen-month-old cynomolgus macaques were immunized with one of the four combinations: two doses of rAd5-Gn (rAd5-Gn/rAd5-Gn); two doses of purified Gn protein (Gn/Gn); rAd5-Gn followed by purified Gn (rAd5-Gn/Gn); and purified Gn protein followed by rAd5-Gn (Gn/rAd5-Gn) at weeks 0 and 2. Heterologous vaccination with rAd5-Gn/Gn induced the most potent humoral immunity characterized as anti-Gn total IgG and SFTSV-neutralizing antibody titer. Furthermore, rAd5-Gn/Gn heterologous vaccination provided balanced immunogenicity between Th1 and Th2 responses demonstrated as IgG1 and IgG2 titer. On the other hand, the rAd5-Gn/Gn combination provided the most robust cellular immunity, as demonstrated by the activation of follicular helper T cells, effector T cells, central memory T cells, and IFN-γ producing T cells.

In an in vivo challenge study, WT C57BL/6 mice were immunized with PBS control, Gn/Gn, rAd5-Gn/Gn, or Gn/rAd5-Gn, and then were injected with IFNAR-NAb and IL-10 cytokine at 24 h prior to 1 × 10^5^ FFU lethal SFTSV infection. Interestingly, only mice immunized with rAd5-Gn/Gn and Gn/rAd5-Gn combinations were protected from body weight loss, splenomegaly, liver damage, and viremia. On the other hand, mice immunized with homologous vaccination (Gn/Gn) displayed significant body weight loss, splenomegaly, viremia, and hepatocytic necrosis. These findings suggest the enhanced immunogenic potential of Gn in a heterologous vaccination approach. Further analyses, such as long-term immunogenicity and pre-existing Ad5 immunity, are needed to characterize the potential of the heterologous vaccination for future clinical vaccine trials.

### 4.7. mRNA Vaccine Platform

mRNA vaccine has demonstrated its potential in combating SARS-CoV-2, attributed to rapid design and scalability for mass production [140,141,142]. However, the mRNA vaccine still needs an improvement in mRNA stability and vaccine storage [143].

As described in Section 4.5.3, Kim et al. also applied SFTSV GnH and GnH-FT antigens for the mRNA vaccine platform [144]. In this study, a linear GnH or GnH-FT DNA template under the control of a T7 promoter, with 5′ and 3′ untranslated regions, was transcribed through in vitro transcription with N1-Methyl-pseudoUTP and CleanCap 5′ cap1 analog, and synthesized mRNAs were subsequently encapsulated into a lipid nanoparticle (LNP). BALB/c mice were intramuscularly immunized with GnH or GnH-FT mRNA-LNP. Mice immunized with GnH or GnH-FT-encoding mRNA-LNPs induced anti-GnH total IgG and SFTSV-neutralizing antibody quantified as IC_50_. Mice immunized with the GnH-FT mRNA vaccine induced stronger anti-GnH IgG and SFTSV-neutralizing antibody responses (up to 2.28-fold) until week 15 than mice immunized with GnH mRNA vaccine.

When IFNAR KO mice immunized with GnH or GnH-FT-encoding mRNA-LNPs were challenged with 2 × 10^4^ PFU of SFTSV, all immunized mice were fully protected from symptoms of body weight loss and fatality upon virus challenge. In contrast, PBS-immunized control mice demonstrated severe body weight loss and 100% fatality by four dpi. These data suggest the strong efficacy of GnH and GnH-FT mRNA vaccines in the mouse pathogenesis model.

A similar outcome was also observed in a recent study by Kim et al., which used SFTSV Gn for their mRNA vaccine development [145]. In this study, WT C57BL/6 mice were immunized with two doses of mRNA encoding SFTSV Gn at weeks 0 and 2. Immunized mice successfully induced humoral and cellular immunity, and a subsequent experiment showed that immunized mice were protected from fatal infection after receiving IFNAR-NAb and the lethal dose of 1 × 10^5^ FFU at week 6. Future studies should be directed not only to characterize the maintenance of humoral and cellular immunity to better understand long-term immunity against SFTSV, but also to discover optimal conditions for vaccine doses, immunization routes, time points, and homologous and heterologous SFTSV challenges in various animal models.

**Table 1:** SFTSV vaccines developed to date. Vaccine candidates with insufficient immunogenicity are italicized. BW stands for body weight (enlarged original table also provided separately).

	Viral Antigen	Carrier/Adjuvant	Dose	Immunization Schedule	Animal Model Used	Viral Challenge	Efficacy	Advantage	Disadvantages	Reference
**Live-attenuated vaccine**	Truncation mutant of NSs	-	4 × 10^6^ pfu (rHB2912aaNSs)	Single immunization	Aged ferrets	10^7.6^ TCID_50_	Full protection from BW loss, fever, serum viremia, fatality	Potent immunogenicity	Concern of reversion mutation and side effect from potentially excessive immunogenicity	[111]
	Substitution mutant of NSs		5 × 10^5^ pfu (rHB29NSsP102A)							
**DNA vaccine**	Gn/Gc		40 μg	Weeks 0, 3	Aged ferrets Immunized & serum-transferred	10^7.6^ TCID_50_, 2 or 4 weeks after the last immunization	Full protection from both immunized & serum-transferred ferrets BW loss, fever, thrombocytopenia, leukopenia, viremia, and fatality	Low cost of production	Concern of genome integration Electroporator required for immunization	[112]
	*Gn/Gc, RdRp, N, and NSs*	-	4 μg	Weeks 0, 2, 4	IFNAR KO mice	1 × 10^5^ ffu 2 weeks after the last immunization	*40% fatality and severe BW loss, thrombocytopenia, and viremia*			[113]
	Gn, Gc, RdRp, N, and NSs	Mouse IL-12					Full protection from BW loss, thrombocytopenia, serum viremia, and fatality			
**Whole inactivated vaccine**	Inactivated whole virion	Al(OH)_3_ adjuvant	Glycoproteins of 0.25, 1, or 4 μg	Weeks 0, 2, 4	Wild type C57BL/6	10^5^ TCID_50_ 2 weeks after the last immunization	Accelerated viral clearance (WT C57BL/6 does not develop SFTS)	Balanced B & T cell activation Replicates natural infection	Logistic difficulty of large culture of BSL-3 agent (SFTSV)	[114]
**Viral vectored vaccine**	Gn/Gc	rVSV	2 × 10^4^ pfu	Single immunization	IFNAR KO mice	2 × 10^4^ ffu 30 days after the last immunization	Full protection from BW loss, viremia, and fatality	Strong induction of B and T cell & Ease of mass production	Potential risk of side effects	[118]
	Gn/Gc	rVACV (from m8 strain)	1 × 10^6^ pfu	Weeks 0, 2	IFNAR KO mice	1 × 10^3^ and 1 × 10^5^ TCID_50_ 2 weeks after the last immunization	Full protection from BW loss and fatality		Reduced immunogenicity from pre-existing immunity	[124]
**Protein subunit vaccine**	*NSs*	*Complete Freund’s adjuvant*	*100 μg*	Weeks 0, 2	*Wild type C57BL/6*	*3* × *10^7^ pfu* *12 days after the last immunization*	*Failed to accelerate viral clearance* *(WT C57BL/6 does not develop SFTS)*	Higher safety profile for the elderly population	*Failed to provide immunity*	[131]
	*Gn*	*Human Fc & Alum adjuvant*	*20 μg*	Weeks 0, 2	*IFNAR KO mice*	*1* × *10^5^ ffu* *2 weeks after the last immunization*	*50% fatality and severe BW loss*		*Failed to provide sufficient immunity*	[113]
	*Gc*						*100% fatality and severe BW loss*		*Failed to provide immunity*	
	Gn (head region)	Ferritin nanoparticle & AddaVax adjuvant	15 μg	Weeks 0, 2, 4	Aged ferrets	1 × 10^7.6^ TCID_50_ 2 weeks after the last immunization	Full protection from BW loss, fever, thrombocytopenia, leukopenia, viremia, and fatality		Cost of production	[132]
**Heterologous vaccine**	Gn (head region & head + stem region)	rAd5 + CrPV & Al(OH)_3_ adjuvant	rAd5-Gn/Gn: 1 × 10^9^ IU rAd5-Gn, then 5 μg Gn	Weeks 0, 2	Wild type C57BL/6 injected with IFNAR-NAb and IL-10	1 × 10^5^ ffu 3 weeks after the last immunization	Full protection from BW loss, splenomegaly, liver damage, and viremia	rAd5-Gn reduces cost of production	Complicated immunization protocol	[139]
			Gn/rAd5-Gn: 5 μg Gn, then 1 × 10^9^ IU rAd5-Gn							
**mRNA vaccine**	Gn (head region)	lipid nanoparticle	3 μg	Weeks 0, 3	IFNAR KO mice	2 × 10^4^ pfu 2 weeks after the last immunization	Full protection from BW loss and fatliaty	Low cost & rapid production	Cold chain supply required	[144]
	Gn (head region)	Ferritin & lipid nanoparticle								

## 5. Conclusions and Future Directions

The emergence of the Asian long horned tick as a carrier of SFTSV beyond east Asia has raised significant public health concerns regarding SFTSV infection. Given its recent emergence, there is currently no targeted antiviral treatment or approved vaccine for SFTSV. Several strategies for developing SFTSV vaccines have been suggested, including live-attenuated virus vaccines, DNA vaccines, whole inactivated vaccines, viral vector vaccines, protein subunit vaccines, and mRNA vaccines. Each vaccine platform possesses distinct advantages and limitations, rendering them more suitable for specific populations or regions. Vaccine platforms tailored for each target subpopulation would further enhance the accessibility and protection of public health in areas with varying clinical and financial resources. Nonetheless, a comprehensive evaluation of immunization protocols and the effectiveness and safety of SFTSV vaccine candidates is imperative for their prospective clinical application, particularly among the elderly population. Additionally, logistical considerations such as manufacturing costs, transportation, and vaccine stability must be taken into account to ensure the safe and efficient development of SFTSV vaccines.

## Data Availability

No new data were created.
